# Effective use of latent semantic indexing and computational linguistics in biological and biomedical applications

**DOI:** 10.3389/fphys.2013.00008

**Published:** 2013-01-30

**Authors:** Hongyu Chen, Bronwen Martin, Caitlin M. Daimon, Stuart Maudsley

**Affiliations:** ^1^Laboratory of Neuroscience, Receptor Pharmacology Unit, National Institute on Aging, National Institutes of HealthBaltimore, MD, USA; ^2^Laboratory of Clinical Investigation, Metabolism Unit, National Institute on Aging, National Institutes of HealthBaltimore, MD, USA

**Keywords:** latent semantic indexing, data mining, computational linguistics, molecular interactions, drug discovery

## Abstract

Text mining is rapidly becoming an essential technique for the annotation and analysis of large biological data sets. Biomedical literature currently increases at a rate of several thousand papers per week, making automated information retrieval methods the only feasible method of managing this expanding corpus. With the increasing prevalence of open-access journals and constant growth of publicly-available repositories of biomedical literature, literature mining has become much more effective with respect to the extraction of biomedically-relevant data. In recent years, text mining of popular databases such as MEDLINE has evolved from basic term-searches to more sophisticated natural language processing techniques, indexing and retrieval methods, structural analysis and integration of literature with associated metadata. In this review, we will focus on Latent Semantic Indexing (LSI), a computational linguistics technique increasingly used for a variety of biological purposes. It is noted for its ability to consistently outperform benchmark Boolean text searches and co-occurrence models at information retrieval and its power to extract indirect relationships within a data set. LSI has been used successfully to formulate new hypotheses, generate novel connections from existing data, and validate empirical data.

## Introduction

Over the past decade the ability for biomedical scientists to generate large-scale data sets has surpassed the processing capabilities of standard analytical tools. The high content and volume of large “*omic*” data sets make identification of key factors and the elucidation of cryptic data connections increasingly problematic. A sensible option for data analysis and information extraction is to preprocess the data to form distinct, functional groups. For many bioinformatic applications, this form of preprocessing is accomplished by clustering genes/proteins into pre-determined Gene Ontology (GO) term groups or canonical signaling pathways, e.g., KEGG (Kyoto Encyclopedia of Genes and Genomes) or BioCarta. However, this data organization relies upon the accuracy and fidelity of experimentally-driven human curation of these groups or pathways. These grouping functions may be artificially exclusive and also potentially outdated by subsequently-obtained experimental data. These systems, while providing an effective form of data analysis, are inherently rigid in their construction and therefore could be supplemented by using alternative strategies, e.g., Latent Semantic Indexing (LSI) or Latent Semantic Analysis (LSA). LSI is a commonly-used dimensionality-reduction technique used to compare similar “concepts/topics” among a collection of terms or documents. LSI is frequently employed in language processing to serve a variety of purposes, e.g., text categorization, indexing, essay grading, image auto-annotation, and automatic cross-language retrieval (Foltz and Dumais, 1992; Dumais et al., 1997; Deerwester et al., 1999; Sebastiani, 2002; Monay and Gatica-Perez, 2003). The utility of LSI stems from its ability to address multiple problems associated with other information retrieval methods: sparseness, noise, term independence, synonymy, and polysemy. Synonymy is defined as two terms conveying the same semantic meaning. Therefore, with a conventional Vector Space Model (VSM), two vectors could be similar even though their similarity lies in values from different dimensions (terms). Conversely, polysemy is defined as the same term having different meanings. Therefore, with a conventional VSM, two identical vectors can theoretically have different meanings. Term independence assumes that one term's presence does not affect any other terms currently in the document.

As the volume of textual information increases in the biomedical field, literature mining is becoming an effective approach to extract physiological meaning from such data sets. The interrogation of well curated bodies of accessible biomedical data, e.g., PubMed and the Gene Expression Omnibus, with LSI/LSA is likely to enhance our appreciation of complex, multifactorial disorders such as Alzheimer's disease. In this review, we will outline the mechanical structure of LSI-based approaches, demonstrate their ability to aid data extraction from mass data sets as well as discuss the relative benefits and drawbacks of such tools in the realm of biomedical data mining.

## Mechanics behind latent semantic indexing

**Figure 1:**
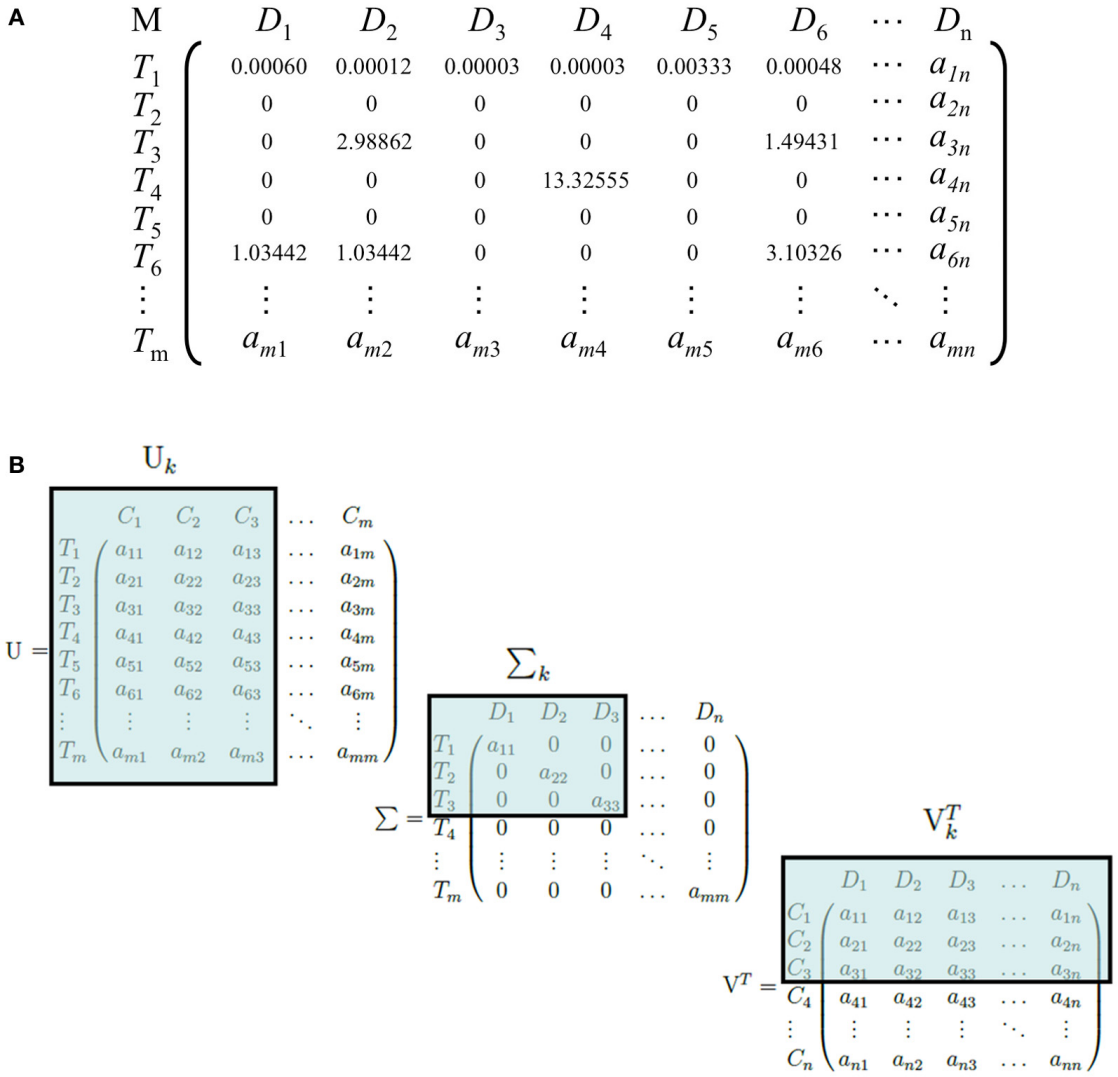
**(A)** An example of a term-document matrix with a weighting function (tf-idf). M, D, and T refer to the term-document matrix, the set of all documents in the corpus, and the set of all terms in the corpus, respectively. T_1_ is an example of a common word that occurs frequently in documents, whereas T_3_, T_4_, and T_6_ are comparatively rarer words and receive a higher weight. **(B)** An illustration of the dimensionality-reduction step of LSI. U, Σ, and V^T^ are truncated and become Σ_k_, U_k_, and V^T^_k_, respectively. C, D, and T refer to the set of LSI topics, documents, and terms, respectively. Here, we illustrate a reduction to three dimensions.

LSI can be used on any corpus involving the use of conceptual identifiers, such as words of any language, identification numbers or letters, indices, morphemes, or any meaningful tokens. A matrix, M, is constructed from the corpus with each row representing the set of all terms, T, and each column representing the set of all documents, D (Figure 1A). Each entry a_ij_ in the matrix is positively defined by a weighting function if T_i_ ∈ D_j_, and zero otherwise. Common weighting functions such as log-entropy, term frequency-inverse document frequency (tf-idf), and term frequency-normal (tf-normal) are used to underweigh common words and overweigh infrequent words that are likely to be more discriminatory identifiers of a document. The resulting matrix is referred to as the “term-document” matrix. An important trait of weighting functions such as tf-idf, tf-normal, and log-entropy is to map a discrete power law distribution, which is exemplified in the vast majority of natural language according to Zipf's law, into a continuous Gaussian function, a requirement for a later step, Singular Value Decomposition (SVD).

Next, SVD is performed on the term-document matrix M (Golub and Reinsch, 1970). Briefly, SVD factorizes the matrix into three matrices: Σ, a diagonal matrix with the square roots of the eigenvalues of MM^T^ sorted in descending order; U, a square matrix of dimensions T × T with each column representing the eigenvector of MM^T^ that corresponds to each eigenvalue in Σ; and V^T^, the transpose of a square matrix of dimensions D × D with each column representing the eigenvector of M^T^M corresponding to each eigenvalue in Σ. For an arbitrary matrix M, there exists at least one factorization into UΣV^T^ via SVD, where the singular value matrix is guaranteed to be unique. The original matrix M can be reconstructed by the equation UΣ V^T^.

The matrix U is the term-concept matrix, with each entry representing a term's relation with a concept. Similarly, V^T^ is the concept-document matrix, with each entry representing a document's relation with a concept. LSI then performs the dimensionality-reduction step by truncating each matrix. The top *k* singular values are taken from Σ, because they capture the most variance from the original matrix, and the first *k* columns and rows are taken from U and V^T^, respectively. The resulting matrices Σ_k_, U_k_, and V^T^_k_ capture the reduced-dimension representation of M (Figure 1B).

These matrices can then be used as a distance metric for both terms and documents. Any two documents can be compared by computing the cosine distance between their corresponding column vectors in V^T^. Likewise, any two terms can be compared by computing the cosine distance between their corresponding row rectors in U. All user generated queries are treated as a separate document. However, SVD does not need to be repeated. Rather, since M = UΣV^T^ and therefore V = M^T^UΣ^−1^, one can index the user query *q* by adding a new column to M with the same weighting function, and right multiply the transpose by U and Σ^−1^ to attain the concept-document matrix. The query can then be compared to all existing documents using cosine distance (Berry et al., 1995).

Because of its use of various linear algebra techniques, LSI possesses many advantages over standard Boolean term searching and VSMs. First, LSI is used in conjunction with, and not instead of, common Boolean search weighting functions such as tf-idf. Term independence, an assumption of the standard VSM, which is false in some applications, is not assumed in LSI. Whereas 99% of all entries in a typical term document matrix are zero, making sparseness a problem, most of the entries in the resulting LSI matrices are non-zero (Landauer et al., 1998). Noise is reduced during the dimensionality-reduction step, since the noise is assumed to be in the discarded columns and rows. LSI addresses synonymy by the fact that synonyms are commonly used in the same context and therefore LSI concepts are likely to reflect them. Polysemy is addressed, though debatably inadequately, by the noise reduction—as infrequent uses of a particular word may be discounted during the dimensionality-reduction step. LSI possesses advantages over other dimensionality reduction techniques such as covariance-based Principal Component Analysis (PCA). The latter performs an eigen-decomposition on the computed (square) covariance matrix, whereas LSI applies SVD directly on the (non-necessarily square) input matrix.

## Enhanced distance metric over conventional models

Despite LSI's widespread usage in linguistics, it remains an underappreciated tool in biology. Often a comparison between two or more articles, genes or proteins is required for the analysis, clustering, categorization, and classification of such entities. The distance metric used for comparison is crucial for determining the quality of the algorithms that employ it. A high quality distance metric must tolerate sparseness, disregard noise and capture the intrinsic and extrinsic links between two entities. As a result, LSI can be used as an effective distance metric, and has been shown to outperform co-occurrence models and simple VSMs (Deerwester et al., 1999; Homayouni et al., 2005; Chagoyen et al., 2006; Klie et al., 2007; Ha et al., 2011; Roy et al., 2011; Xu et al., 2011). LSI's enhanced distance metric stems from its robustness against noise, synonymy and polysemy due to reduced dimensionality.

A basic application of this distance metric is the measure of similarities among clinical documents. As previously mentioned, LSI is not dependent upon specific languages or grammars. Ha et al. applied LSI to a corpus of Korean discharge summaries and newspaper articles and noted that LSA-measured document similarities correlated with co-occurrence and was effective at measuring both Korean lexical morpheme-to-morpheme and document-to-document similarities (Ha et al., 2011). Using LSI's freedom from the necessity of grammatically-correct English language, biologists have frequently employed “gene documents” to a concatenation of all MEDLINE abstracts associated with a specific gene. “Gene documents” allow biologists to measure the similarity between two genes by mining the biomedical literature associated with each gene. LSI can be applied to these documents and all pairwise distance metrics among genes used for a variety of purposes, including agglomerative hierarchical clustering, determining the “cohesion” of a gene list and identifying transcription factor candidates (Homayouni et al., 2005; Roy et al., 2011; Xu et al., 2011). These tasks' precision and recall were evaluated on a “gold standard” set and deemed to outperform that obtained by benchmark co-occurrence methods. Similar results have been obtained for proteins (Chagoyen et al., 2006; Klie et al., 2007).

## Latent links for literature-based biomedical discovery

**Figure 2:**
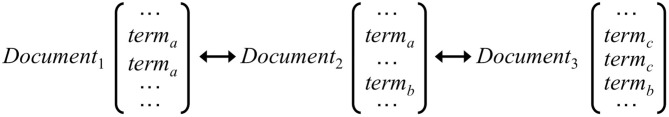
**An illustration of a “latent” link between *term*_*a*_ and *term*_*c*_ because of their respective co-occurrence with *term b*.** The principle of Swanson discovery is analogous to this—we have two currently disjointed sets of literature A and C and bridge the gap by introducing an intermediate literature set B.

Literature-based discovery describes the problem of extracting previously unknown connections in two disjoint sets of scientific literature through the use of an intermediate set (Swanson, 1987, 1989, 1990). LSI's decreased dependence on direct term matches allows for the extraction of hidden relationships among concepts. For example, a hidden link can occur between the concepts denoted by term A and term C because of their respective co-occurrence with term B, even though they do not co-occur themselves (Figure 2). This relationship, on the term level at least, is the core principle of Swanson discovery. Therefore LSI has been shown to be a powerful tool in identifying potential discoveries from the scientific literature without *de facto* empirical scientific demonstration of a direct linkage (Gordon and Dumais, 1998).

Kim et al. attempted to retrieve unrecognized gene relationships by using LSI along with Non-Negative Matrix Factorization (NMF), another matrix factorization method (Kim et al., 2007). Gene retrieval was evaluated on manually created test sets based on precision and recall, showing that LSI- and NMF-based methods vastly outperformed co-occurrence methods. Similarly, Roy et al. demonstrated LSI's ability to identify implicit links between transcription factors derived from a set of differentially expressed genes (Roy et al., 2011). ComputableGenomix's web-based semantic search engine, GeneIndexer, uses LSI on MEDLINE abstracts to identify known and unknown gene relationships. Typically, strongly correlated factors demonstrate cosine similarities in the document matrices near 0.4–0.7 while implicit associations are only considered valid for further investigation with a cosine score of >0.1 (Homayouni et al., 2005; Roy et al., 2011). Using this discovery type of dataset interrogation biologists have been able to identify promising yet previously unknown links between genes and user-defined input words (Lee et al., 2007; Tijoe et al., 2008; Chadwick et al., 2011a).

## Validation of observations against current literature

LSI-based tools allow for the validation of experimental observations. Statistically significant differences amongst gene-keyword associations between experimental and control groups, using relevant keywords, can illustrate whether empirical observations are well-supported by the scientific literature. For example, Chadwick et al. used GeneIndexer to demonstrate that a much larger proportion of significant gene-keyword interactions existed in the Alzheimer's disease mouse model (3×TgAD) group compared to the control group (Chadwick et al., 2010a). With experimentally relevant keywords like “Alzheimer's” and “oxidation,” this finding coincided with experimental results, lending support to the experimental observations. Similar experiments have also used GeneIndexer as validation (Chadwick et al., 2010b, 2011b,c, 2012; Zhou et al., 2011). Using this LSI-based approach, accurate data “phenotypes” can be generated by using protagonistic and antagonistic gene-keyword combinations (Chadwick et al., 2010b). Therefore, a well-informed user can generate a gestalt appreciation of the potential functional inter-relationships of all of the genes/proteins in the original data set. Wei et al. used an LSI-based transcriptional factor identification method to validate the role of cRel as a regulator of interferon-simulated genes (Wei et al., 2008). One important aspect of literature mining is that the source of validation is constantly evolving. Literature-based discovery and LSI-based validation generate new scientific discoveries, which, when published in biomedical databases like PubMed, can be indexed again for future analysis.

## Visualization of high-dimensional data

LSI can be used to enhance visualization of data in two ways. First, it allows for extraction of information from unstructured or semi-structured corpora. LSI, in conjunction with other natural language processing techniques, can be used to interpret key concepts from a corpus and project it back to the user in graphical form. Jahiruddin et al. implemented this concept by creating BioKEVis, a search interface that produces semantic nets for the visualization of biomedical knowledge from PubMed (Jahiruddin et al., 2010). Second, LSI's ability to reduce dimensionality allows for a better visualization of high-dimensionality points that exceed the realm of physical space. For example, LSI can be used to reduce the number of dimensions in vector space to one, two, or three so that each point is graphable in three-dimensional space (Kim et al., 2007). A major disadvantage to this method is that three dimensions is typically not an optimal value for *k*, so information loss will be significant. To maintain performance, dimensionality-reduction to an optimal *k* can be performed to reduce noise, and then various high-dimensionality visualization techniques can be used to visualize the resulting, lower-dimensionality data (Swayne et al., 1992, 1998). With more accessible visualization of data, users can form their own interpretations of the data in addition to what has been presented by algorithmic analysis.

## Limitations of LSI-based analyses

Though undoubtedly a useful tool, LSI does possess some disadvantages. The most obvious disadvantage is the selection of *k*, or the number of vectors in U and V^T^ to keep. A high *k* value may seem advantageous because one compares all documents across more concepts, but can be detrimental due to added noise. Conversely, a low *k* value suffers from the danger of discarding crucial, distinguishing concepts in the data. This problem can be ameliorated to a certain extent by optimizing the precision and recall of LSI retrieval with *a priori* knowledge (Dumais, 2004; Kim et al., 2007). Analysis of the variance captured by the current dimensions, similar to that of PCA, is another method frequently employed (Cangelosi and Goriely, 2007). Overall, anywhere between 300 and 500 is appropriate for large corpora of millions of documents (Bradford, 2008). Another limitation of LSI is that it is computationally intensive. Calculating the SVD of a matrix M via reduction to a bidiagonal matrix has a computational complexity of O [*m* × *n* × min (*m*, *n*)], where *m* and *n* are the number of rows and columns in M, respectively. For large term-document matrices, such computation is unfeasible. However, since only the reduce-rank matrix of the SVD of M is used for LSI, one can perform “rank-reduced” SVD on M, yielding a computational complexity of O (*m* × *n* × *k*), which is more scalable (Jahiruddin et al., 2010). In addition, along with high *k* values and inherent computational complexities, the future application of LSI to biomedical data may be hampered by the ever-increasing need for expanded data storage space. Finally, LSI uses the bag-of-words model when converting a corpus into the term-document matrix. The ordering of words in a document is completely disregarded, even though it is undoubtedly important. Despite this, there have been efforts to incorporate grammatical relations, sentence structure, and parts-of-speech tagging into LSI for biological fields (Klein and Manning, 2003; Brand, 2005; Settles, 2005).

## Integration of LSI with classical informatics

With LSI-based information retrieval it is now possible to detect undiscovered molecular interactions. Even though standard data clustering/enrichment processes can only aid the interpretation of existing data, we cannot consider these approaches redundant. “Combinatorial informatics” comprises a synergistic combination of both LSI with standardized bioinformatic workflows. We have recently developed such a workflow to facilitate the discovery of biomolecular “keystone” factors (Chadwick et al., 2012). Mathematical modeling of “real-world” networks, has demonstrated that complex systems are not connected in an equitable and homogenous manner. Network connections can occur within small, tightly-connected “small-world networks” or between different “small-world networks” (Watts and Strogatz, 1998). From a biological standpoint, these “small-worlds” are analogous to biological processes such as kinase signaling cascades, while components of endocrine or neuronal axes could represent the constellations of these groups of small-world networks. Within global networks of genes/proteins, there are likely to exist specific genes/proteins that form the most important bridges between multiple “small-world” networks. Such genes/proteins within a functional network are often described as keystones. Keystones enhance rapid connectivity between disparate parts of a network and, as such, can be considered as functional “short-cuts” within the system (Watts and Strogatz, 1998). It has been shown that that even in networks commensurate with the biological scale (containing thousands to millions of nodes), surprisingly few (5–10) “short-cuts” are required to facilitate rapid information transfer across large systems (Watts and Strogatz, 1998). Classical KEGG/GO data set enrichment analysis can be transferred into LSI-based queries to assist in the discovery of keystone factors (genes/proteins) that possess a disproportionate ability to associate with the greatest number of the predicted KEGG/GO signaling paradigms.

## Conclusion

LSI has been successfully employed in a variety of biological contexts from the clustering of gene sets to the visualization of high-dimensionality data. Its ability to alleviate the effects of sparseness and noise, common traits of high-throughput “*omics*” data, makes textual analysis possible on data sets where standard term searching produces inadequate results. LSI is independent of the constraints of specific languages or grammars, thereby allowing researchers to employ gene documents, protein/experiment documents, and even noun-phrase documents to address the problem at hand. LSA, in the field of linguistics, has seen a wide variety of suggested improvements over the years. Probabilistic variants such as Probabilistic LSA and Latent Dirichlet Allocation have been suggested for their addition of a more accurate probabilistic model with respect to understanding of semantic concepts (Hofmann, 1999; Blei et al., 2003). Additional variants, including Hierarchical Dirichlet Processes and Random Projections, are interesting alternatives to LSI, that accomplish the same goals of dimensionality-reduction and topic modeling (Gionis et al., 1999; Teh et al., 2006). These methods, while not better or worse are certainly viable alternative candidates for biological data mining that should be evaluated alongside LSI. There seems however to be an unfortunate “lag” between developments in LSI and its integration with biomedically-related fields. For instance, PubMed was initially released in 1996, 6 years after the development of LSI. However, it was not until 2009 that PubMed released a searching algorithm not dependent upon outdated Boolean term searches. The most recent, state-of-the-art developments in computational linguistics and LSI/LSA may however require years, or even decades, to be accepted and used widely in the biological community. Nevertheless, with a conscious effort to improve data quality for literature mining with the use of standardized terms (MeSH, KEGG, GO), text mining is becoming increasingly viable and popular (Ashburner et al., 2000; Coletti and Bleich, 2001). With a realization of the importance of inter-disciplinary analysis and increased collaboration between biologists and computational linguists, there is the exciting possibility of rapid advancement in the field of literature mining as an important bioinformatics technique.

### Conflict of interest statement

The authors declare that the research was conducted in the absence of any commercial or financial relationships that could be construed as a potential conflict of interest.
